# Updated beliefs and shaken confidence: evidence from vaccine hesitancy caused by experiencing “COVID arm”

**DOI:** 10.1186/s12879-023-08558-5

**Published:** 2023-09-18

**Authors:** Taiyo Fukai, Keisuke Kawata, Masaki Nakabayashi

**Affiliations:** 1 Faculty of Humanities and Social Sciences, University of Tsukuba, Tsukuba, Ibaraki Japan; 2 Institute of Social Science, The University of Tokyo, Bunkyo, Tokyo Japan

**Keywords:** Belief updates, “COVID arm” symptoms as an exogenous shock, Confidence in vaccination, Acknowledged importance of vaccination, Confidence in science

## Abstract

**Background:**

Public health depends largely on people’s knowledge, beliefs, or behaviors regarding their health and medical treatments. Although works based on the health belief model have shown that public beliefs about medical treatments affect willingness to take the treatments, little is known about the effects of changes in beliefs on attitudes toward treatment. How one’s past experiences relate to one’s beliefs about a given medical treatment is worth considering.

**Methods:**

We implemented an online panel survey in February 2021 and March 2022 in Japan before and after COVID-19 vaccines were administered to the public within the country. We exploited delayed localized hypersensitivity reactions to COVID-19 vaccines, namely, “COVID arm”, as an exogenous shock to investigate the relationship between past negative experiences and current beliefs about medical treatments or science. “COVID arm” was an unexpected side effect and thus likely caused updated beliefs about the vaccine. Out of the nonprobability sample of 15,000 respondents in the first wave in February 2021, 9,668 respondents also responded to the second wave conducted in March 2022. Outcome variables were whether experiencing “COVID arm” affected the respondents’ 1) confidence in vaccine safety, 2) willingness to take the next dose of COVID-19 vaccines, 3) acknowledgment of the importance of vaccination, and 4) confidence in science. We measured the impact of experience with “COVID arm” on changes in the probability that survey respondents would respond affirmatively to questions posed about the issues listed above.

**Results:**

Experiencing “COVID arm” significantly lowered confidence in the safety of vaccination by 4.3 percentage points, which was approximately 6% of the sample mean for the first wave, and lowered the probability of taking a second dose of the COVID-19 vaccine by 1.5 percentage points. These adverse impacts were observed after conditioning background characteristics and prior confidence in vaccination. Experiencing “COVID arm” affected neither the acknowledged importance of vaccination nor confidence in science in a statistically significant way.

**Conclusions:**

An unexpected and uncomfortable shock regarding beliefs about a treatment decreases willingness to take the treatment. An appropriate public health policy should account for this effect.

**Trial registration:**

The survey was preregistered with the American Economic Association’s RCT Registry (Fukai et al., 2022).

**Supplementary Information:**

The online version contains supplementary material available at 10.1186/s12879-023-08558-5.

## Introduction

General public confidence in technologies and the perceived risk of technologies are associated [1]. Similarly, beliefs about specific medical treatments affect willingness to take the treatments, as demonstrated by works based on the health belief model. The health belief model classifies beliefs related to a treatment into several dimensions, such as perceived benefits and perceived barriers, and helps in investigating the impact of each dimension on willingness to take the treatment [2, 3, 4, 5, 6, 7, 8, 9]. Thus, in addition to the development process of medical treatments itself, public confidence in the developed medical treatments is essential to public health because it affects the public’s willingness to take the treatments. Public willingness to take a medical treatment is likely to depend on beliefs about the benefits and risks of the treatment. Events that alter such beliefs might affect the public’s confidence in the medical treatment. However, such changes are hard to observe systematically under ordinary circumstances. This makes it difficult to identify the causality of certain events regarding belief updates about a medical treatment and changes in confidence in the medical treatment.

The COVID-19 pandemic has provided unusual circumstances in which we can test hypotheses about such a causal relationship between belief updates about a medical treatment and a change in confidence in that treatment. First, since COVID-19 vaccines were a new treatment for everyone, the uncertainty surrounding the related effects and side effects was higher [10]. Additionally, higher expected probabilities of side effects of COVID-19 vaccines were expected to discourage vaccination [11–25]. Second, a substantial subset of recipients experienced delayed localized hypersensitivity reactions to COVID-19 vaccines [26, 27]; such reactions, collectively referred to as “COVID arm”, have been objectively observed. Moreover, conditional on possible confounders such as the recipients’ background, prior confidence in vaccine safety, prior confidence in the vaccine licensing authority, and prior vaccine hesitancy, we can identify whether “COVID arm” symptoms changed, through belief updates about the vaccine’s side effects, general confidence in vaccine safety, confidence in the vaccine licensing authority, acknowledgment of the importance of vaccination, and confidence in science in general as causal relationships.

Experiencing a dose of new vaccine might update beliefs about the vaccine. Notably, unexpected consequences might change beliefs. To investigate whether an unexpected and uncomfortable experience with vaccination has affected the public’s confidence in vaccine safety, authorities’ licensing process for vaccines, and science in general through belief updates, we conducted a panel survey in Japan in February 2021, when COVID-19 vaccines were not yet administered to the public in Japan, and in March 2022, when the first and second doses were available to everyone, as described in Progress in COVID-19 vaccination section. In the first and second waves, we asked the same questions regarding confidence in vaccine safety and the vaccine licensing authority in Japan. In the second wave, we also asked the respondents about 1) whether they took the first and second doses of COVID-19 vaccines and whether they had taken or wanted to take the third dose, which captures the degree of vaccine hesitancy; 2) whether they experienced “COVID arm” symptoms; and 3) what level of confidence they had in science in general.

The novelty of our study is first that we asked the same respondents about their beliefs regarding vaccination both before and after being vaccinated against COVID-19. Second, we exploited a side effect of vaccination, “COVID arm” symptoms, as an exogenous shock to investigate the relationship between past negative experiences and current beliefs about medical treatments and science.

## Our aim

It has been reported that beliefs about vaccines, authority, and science are associated with confidence in vaccine safety and willingness to be vaccinated. Using panel data from Germany, Schmelz and Bowles showed that factors associated with support for mandated COVID-19 vaccination include beliefs about the efficacy of the vaccines and trust in public institutions [28]. Using data from the UK, Freeman et al. and Veli et al. found that exposure to information about vaccine efficacy reduces vaccine hesitancy [29, 30]. By data from Bosnia and Herzegovina, Aksentijevic et al. reported that positive beliefs about efficacy of CVOVID-19 vaccines were associated with willingness to take up COVID-19 vaccines [31]. Using Southeast Asian data, Duong and Antriyandarti found that respondents who were more informed about vaccine efficacy were less vaccine hesitant [32]. Denford et al. found from interviews in the UK and Gardner et al. found from a survey in the US that vaccine hesitancy was associated with lower trust in the government [33, 34]. Using extensive panel data from 12 countries, Algan et al. found that confidence in science is significantly associated with attitudes toward vaccinations [35]. Using data from 138 countries, Eichengreen et al. found that exposure to epidemics at young ages was associated with lower levels of trust in science and led to lower compliance with protective behaviors against COVID-19 [36]. Using data from the US, Bagasra et al. demonstrated that greater trust in the scientific community predicts lower vaccine hesitancy [37]. Collecting data from Japan, Sekizawa et al. found that a higher level of generalized trust leads to lower vaccine hesitancy [38]. As a caveat, using Ethiopian data, Hassen et al. established that greater confidence in traditional remedies was associated with higher vaccine hesitancy [39]. In summary, trust in modern science or vaccination is associated with a reduction in vaccine hesitancy.

Previous works, represented by Schmelz and Bowles, leave two layers of challenges. One layer concerns what affects beliefs about medical treatments. Although Schmelz and Bowles found associations between beliefs about vaccine efficacy and support for mandated vaccination policy, determinants of such beliefs are beyond their framework. The other layer of challenge relates to possible reverse causality. That is, as Schmelz and Bowles acknowledged, those determined to oppose vaccination might “unwittingly” decide to distrust public institutions and deny the efficacy of vaccination [8, 28]. Our framework addresses these concerns. We use “COVID arm” symptoms as an unexpected and exogenous shock to beliefs about the vaccines’ side effects and then examine whether the belief updates affect confidence in vaccine safety, confidence in the vaccine licensing authority, and vaccine hesitancy.

Thus, our research question was whether experiencing an unexpected side effect from COVID-19 vaccines, which implies an update to one’s belief after experiencing the vaccine’s side effects, affected confidence in vaccine safety. To identify the causal effects of experiences that can alter beliefs about vaccines on confidence in vaccine safety, we used the occurrence of “COVID arm” symptoms as an exogenous shock to beliefs about COVID-19 vaccines. Additionally, differing from the approach by Schmelz and Bowles, we directly estimate causal effects from belief updates about the side effects of vaccination on confidence in the safety of vaccination and willingness to take a next dose of a vaccine rather than on support for mandated versus voluntary vaccination. Therefore, our contribution is directly identifying a causal relationship between an unexpected and uncomfortable experience through belief updates about vaccination side effects and a change in vaccine hesitancy. The survey was preregistered with the American Economic Association's Randomized Controlled Trials Registry [40].

## Method

### Panel survey

In the first and second waves, we recruited a nonprobability sample of 15,000 Japanese adults through a survey company, Rakuten Insight Ltd., a subsidiary of Rakuten Group, Inc. Respondents were solicited by Rakuten Insight and were rewarded with a certain number of points that could be used for shopping in an e-commerce market, Rakuten Ichiba, offered by Rakuten Group.^1^ The first wave took place from February 17, 2021, to March 4, 2021, and the median response time was 7 minutes. The second wave took place from March 7, 2022, to March 22, and the median response time was 8 minutes. Out of the 15,000 respondents in the first wave, 9,668 also participated in the second wave, and this group formed our final sample.

In both waves, we asked respondents about their confidence in vaccine safety and the vaccine licensing authority’s process and about their recognition of the importance of vaccination. In both waves, we also asked about vaccine hesitancy, namely, whether they had previously taken vaccines other than the COVID-19 vaccines, whether they had previously postponed vaccines suggested by doctors, or whether they had previously refused vaccines, following categorization by MacDonald et al. [41]. In the second wave, we also asked about their level of confidence in science. We followed Eichengreen et al. to create questions on confidence in science [36].

In the second wave, we asked whether the participants had taken the first and second doses of the COVID-19 vaccine, whether they had already taken a third dose of the vaccine and if not, whether they wanted to do so, and whether they had experienced “COVID arm” symptoms. We also asked about their confidence in science and scientists in general.

In both waves, we asked about the following demographic, socioeconomic, and political characteristics: age, gender, marital status, number of children, gender and relative age of siblings, prefecture of residence, working status, highest educational degree, personal income, household income, party support, self-perceived degree of right-leaning political beliefs, degree of dissatisfaction with current politics, and self-perceived social status. In the second wave, we also asked whether the participants had any chronic diseases. Section SA6 of the supplemental appendix presents an English translation of our questionnaire. To estimate the effects of “COVID arm” symptoms, we weighted our sample by sex, age, and employment status background pursuant to the composition of the latest population census in 2020.^2^

### A natural experiment conditional on background characteristics and prior beliefs

After studying the data we collected, we found that the occurrence of “COVID arm” symptoms was associated with certain background characteristics, as presented in Fig. A2 in Section SA2 of the supplemental appendix. Moreover, Fig. A2 also demonstrates that experiencing “COVID arm” symptoms was also associated with prior general confidence in vaccine safety, prior confidence in the vaccine licensing authority, and previous behaviors of vaccine hesitancy. Therefore, we need to balance all factors, including demographic, socioeconomic, and political background characteristics, prior general confidence in vaccine safety, prior confidence in the vaccine licensing authority, and vaccine hesitancy, as described in Balancing covariates section.

In summary, our data indicated that “COVID arm” symptoms and beliefs about vaccine safety, beliefs about the vaccine licensing authority in Japan, and beliefs about science in general were conditionally independent within individuals between the two waves given the background characteristics, prior general confidence in vaccine safety, prior confidence in the vaccine licensing authority, and prior vaccine hesitancy. Therefore, once controlling for background characteristics, prior confidence in vaccine safety, prior confidence in the vaccine licensing authority, and prior vaccine hesitancy, we can treat “COVID arm” symptoms as a natural experiment that randomly dropped in participants [42]. We use this natural experiment to identify a causal relationship between an unexpected and uncomfortable experience with vaccination through belief updates about the side effects of vaccination and confidence in vaccine safety.

Note that it is not a weakness of our design but rather a strength that our data include respondents who were heterogeneous in terms of prior general confidence in vaccine safety, prior confidence in the vaccine licensing authority, and prior vaccine hesitancy. Under our panel design, we can trace both individuals who were confident in vaccination before experiencing “COVID arm” symptoms and those who were skeptical about it. We observed what happened to not only those who were confident in vaccination but also those who were skeptical about vaccination when experiencing “COVID arm” symptoms.

### Treatment and predicted outcomes

Between the first wave in February 2021 and the second wave in March 2022, some of the vaccine recipients experienced “COVID arm” symptoms. We hypothesized that the occurrence of “COVID arm” symptoms changes beliefs about the side effects of COVID-19 vaccines and that downward belief updates would reduce the willingness to take the next dose of COVID-19 vaccines. Our theoretical inference behind this conjecture is straightforward. People decide whether to take a treatment depending on its costs and benefits. An increase in the probability of preventing infection and a reduction in the probability of severe symptoms if infected are benefits of vaccination. Adverse side effects such as “COVID arm” are costs of vaccination. The unexpected experience of “COVID arm” implies an unexpected rise in the cost of vaccination. It leads to a reduction in the relative benefits of vaccination relative to the costs of vaccination. Thus, it might adversely affect the willingness to take COVID-19 vaccines. Therefore, our conjecture flow was as follows.

1. Some people experience the unexpected and uncomfortable symptom of “COVID arm” due to COVID-19 vaccination, but others do not.
2. Experiencing “COVID arm” changes beliefs about the side effects of the COVID-19 vaccines among individuals who were previously confident in vaccine safety and those who were not.
3. Balancing background characteristics, prior general confidence in vaccine safety, prior confidence in the vaccine licensing authority, and prior vaccine hesitancy, we can estimate how experiencing “COVID arm” change, through belief updates, individuals’ confidence in vaccine safety.

Thus, we predicted that “COVID arm” symptoms would affect beliefs about vaccine safety and that the belief updates would affect confidence in vaccine safety, the vaccine licensing authority, and science in general.

### Identification and estimation strategy

Consider potential outcome $Y_{v^{l}, i}\left( D_{i}\right)$ regarding confidence in vaccination, where $D_{i}$ denotes whether the person experienced “COVID arm” symptoms such that

1 $$\begin{aligned} D_{i}=\left\{ \begin{array}{lc} 1 &{}\ \text {if respondent}\ i\ \text{experienced}\ \mathrm{``}\text{COVID arm''}\ \text{symptoms},\\ 0 &{}\ \text {otherwise}.\\ \end{array} \right. \end{aligned}$$

For $$Y_{v^{l}, i}\left( D_{i}\right)$$, $$v^l$$ denotes

$$v^{1}:$$
whether respondent *i* considered vaccination in general to be safe,
$$v^{2}:$$
whether respondent *i* was confident in the vaccine licensing process by the Japanese authority, the Ministry of Health, Labour and Welfare,
$$v^{3}:$$
whether respondent *i* acknowledged the importance of vaccination in general,
$$v^{4}:$$
whether respondent *i* took a second dose of the COVID vaccine,
$$v^{5}:$$
whether respondent *i* took or wanted to take a third dose of the COVID vaccine,

such that

2 $$\begin{aligned} Y_{v^{l}, i}\left( D_{i}\right) =\left\{ \begin{array}{lc} &{}1 \quad \text {if respondent}\ i\ \text{answers}\ \mathrm{``}\text{yes''},\\ &{}0 \quad \text {otherwise,}\\ \end{array} \right. \end{aligned}$$

for $v^{1},..., v^{5}$. Having taken a second dose ($v^{4}$) and having taken or wanting to take a third dose ($v^{5}$) are used to measure posterior vaccine hesitancy after taking the first dose.

As a supplementary survey, we asked the respondents about their confidence in science in general in the second wave such that

3 $$\begin{aligned} Y_{s, i}\left( D_{i}\right) =\left\{ \begin{array}{ll} &{}1 \quad \text {if respondent}\ i\ \text {was strongly confident in science},\\ &{}2 \quad \text {if respondent}\ i\ \text {was moderately confident in science},\\ &{}3 \quad \text {if respondent}\ i\ \text {was not very confident in science},\\ &{}4 \quad \text {if respondent}\ i\ \text {was not confident in science at all.} \end{array} \right. \end{aligned}$$

Then, we obtained three outcome variables such that

4 $$\begin{aligned} Y_{s^{1}, i}\left( D_{i}\right)= & {} \left\{ \begin{array}{ll} 1 &{} \text {if} \ \ Y_{s, i}\left( D_{i}\right) =1,\\ 0 &{} \text {otherwise}, \end{array} \right. \nonumber \\ Y_{s^{2}, i}\left( D_{i}\right)= & {} \left\{ \begin{array}{ll} 1 &{} \text {if} \ \ Y_{s, i}\left( D_{i}\right) \le 2,\\ 0 &{} \text {otherwise}, \end{array} \right. \nonumber \\ Y_{s^{3}, i}\left( D_{i}\right)= & {} \left\{ \begin{array}{ll} 1 &{} \text {if} \ \ Y_{s, i}\left( D_{i}\right) \le 3,\\ 0 &{} \text {otherwise}. \end{array} \right. \end{aligned}$$

Thus, the value of our interest is the expected difference in marginal means between having experienced “COVID arm” symptoms, $D_{i}=1$ and not having experienced such symptoms, $D_{i}=0$ such that

5 $$\begin{aligned} \tau \left( \varvec{x}\right) =E\left[ Y_{j, i}\left( 1\right) -Y_{j, i}\left( 0\right) |\varvec{X}_{i}=\varvec{x}\right] , \end{aligned}$$

where $j \in \{v^{1}, v^{2}, v^{3}, v^{4}, v^{5}, s^{1}, s^{2}, s^{3}\}$ and $\varvec{X}_{i}$ denotes the background characteristics, prior general confidence in vaccine safety, prior confidence in the vaccine licensing authority, and prior vaccine hesitancy of respondent *i*.

As discussed in A natural experiment conditional on background characteristics and prior beliefs section, “COVID arm” symptoms $D_{i}$ and confidence in vaccine safety, the vaccine licensing authority in Japan, and science $Y_{j, i}$ satisfy the unconfoundedness assumption conditional on background characteristics, prior general confidence in vaccine safety, prior confidence in the vaccine licensing authority, and prior vaccine hesitancy $\varvec{X}_{i}$ such that

$$\begin{aligned} D_{i} \perp \!\!\!\perp \left[ Y_{j, i}\left( 0\right) , Y_{j, i}\left( 1\right) \right] \big {|}\varvec{X}_{i}. \end{aligned}$$

Therefore, we identified Eq. (5) as a causal effect of “COVID arm” symptoms $D_{i}$ on posterior general confidence in vaccine safety ($Y_{v^{1}, i}$), posterior confidence in the vaccine licensing authority ($Y_{v^{2}, i}$), posterior acknowledgment of the importance of vaccination ($Y_{v^{3}, i}$), posterior vaccine hesitancy, ($Y_{v^{4}, i}$ and $Y_{v^{5}, i}$), and posterior confidence in science ($Y_{s^{1}, i}$, $Y_{s^{2}, i}$, and $Y_{s^{3}, i}$), given $\varvec{X}_{i}$.

We wanted to obtain the average treatment effect characterized by Eq. (5) as the augmented inverse probability weighting (AIPW) with the double/debiased supervised machine learning algorithm [43–46]. All nuisance functions are estimated by a causal forest algorithm [47]. We conditioned all characteristics, including prior confidence in vaccine safety, prior confidence in the vaccine licensing authority, prior acknowledgment of the importance of vaccination, and prior vaccine hesitancy.

Before analyzing, we weighted our nonprobability sample by demographic characteristics pursuant to the latest population census in 2020, as mentioned above. Then, to control for confounders, the AIPW estimation with supervised machine learning has an obvious advantage regarding robustness against mis-specifications of the model for estimation. Traditional estimation methods such as OLS potentially depend on the specification of the estimation model. A slight misspecification in modeling might result in a substantial bias in estimation. Our approach allows us to semiparametrically estimate the average effect with asymptotical guarantees of debiasedness.

Note that this approach does not require parametric assumptions on the outcome distribution. If we have correct knowledge about the distribution, we may obtain more efficient estimators. However, in our design of the experiment, such knowledge is not available. Thus, we decided that AIPW estimation with supervised machine learning is the most relevant method compared to other possible candidate estimation methods.

In reality, experiencing “COVID arm” symptoms can be associated with prior confidence in vaccine safety, prior confidence in the vaccine licensing authority, prior acknowledgment of the importance of vaccination, and prior vaccine hesitancy, as presented in Table A2 in Section SA2 of the supplemental appendix. We control for all such variables in the AIPW estimation.

Finally, we regressed the AIPW score function on $\varvec{X}_{i}$ by OLS to obtain the best linear projection of $E\left[ \tau \left( \varvec{x}\right) \right]$. All algorithms are included in the *grf* package for R [48].

By balancing the sample with AIPW, we can observe the effects of belief updates due to “COVID arm” symptoms on both those who were confident in vaccination and those who were skeptical about vaccination before experiencing “COVID arm” symptoms. With our panel design, we can estimate the average treatment effects of “COVID arm” symptoms across the groups.

## Descriptive statistics

### Progress in COVID-19 vaccination

The status of vaccination in Japan is summarized as follows. First, Pfizer’s vaccine (BNT162b2) was approved by the Ministry of Health, Labour, and Welfare on February 14, 2021, and the priority vaccination of healthcare workers and others began on February 17, 2021. On April 12, 2021, the prioritized vaccination of approximately 36 million older adults began; at this time, vaccination by municipal governments also began. The ministry approved the Moderna (mRNA-1273) and AstraZeneca (ChAdOx) vaccines on May 21 of the same year. The first round of vaccination started in earnest around May 2021, with the number of daily doses peaking between July and August of the same year. According to the Vaccination Record System, Digital Agency, Government of Japan, the first dose intake rate from February 17 to March 4, 2021, the time of the first survey, was almost 0%. The first intake rate at the time of the second survey, from March 7 to March 22, 2022, was just under 80%.^3^

### Overview

Descriptive statistics of our sample’s background characteristics, prior general confidence in vaccine safety, prior confidence in the vaccine licensing authority, and prior vaccine hesitancy $\varvec{X}_{i}$ for $i=1\ldots 9,668$ are presented in Table A1 in the supplemental appendix. Out of 9,668 respondents who participated in both waves, the 8,321 respondents who received a first dose formed our primary sample. Out of 8,321 respondents, 30 in total did not answer the question about their employment status. Since the information was necessary to weight our sample by sex, age, and employment status pursuant to the composition in the latest population census in 2020, we dropped these 30 respondents and analyzed the remaining 8,291 respondents.

The Japanese government supplied both the Pfizer (BNT162b2) and Moderna (mRNA-1273) vaccines for its domestic vaccination program during the sample period. In our sample, recipients of both the Moderna (mRNA-1273) and Pfizer (BNT162b2) vaccines reported experiencing “COVID arm” symptoms. The self-reported “COVID arm” symptoms in our sample thus included both the severe symptoms triggered often by the Moderna (mRNA-1273) vaccine and less severe symptoms triggered by the Pfizer (BNT162b2) vaccine.

Respondents who had received a first dose of a COVID-19 vaccine as of March 2022 accounted for 86.0% of our sample, those who received a second dose accounted for 84.0%, and those who received or wanted to take a third dose accounted for 32.2%. Among those who did not experience “COVID arm” symptoms during the first dose, 98% took a second dose; among those who experienced “COVID arm” symptoms during the first dose, 97% took a second dose. Furthermore, 38% of those who did not experience “COVID arm” symptoms took or wanted to take a third dose, while 37% of those who experienced “COVID arm” symptoms took or wanted to take a third dose, as of February 2022. Therefore, experiencing “COVID arm” symptoms was associated with a reduction in the uptake of a second dose and the uptake of or willingness to take a third dose by 1 percentage point each time.

### Balancing covariates

Figure A1 of Section SA2 in the supplemental appendix presents a regression of the probability of experiencing “COVID arm” symptoms on the four primary background characteristics: age, gender, vaccine type, and chronic disease status. As reported [26, 27, 49], experiencing “COVID arm” was strongly associated with taking the Moderna (mRNA-1273) vaccine. Additionally, female respondents were more likely to report “COVID arm” symptoms than male respondents, and younger people were less likely to report such symptoms than older people, as has been reported [50, 51]. Having a chronic disease showed a positive but small association with experiencing “COVID arm” symptoms. Furthermore, as Fig. A2 in Section SA2 of the supplemental appendix shows, after the sample was adjusted according to these four covariates, associations with “COVID arm” symptoms were not limited to four primary background covariates. Instead, associations with “COVID arm” symptoms were observed across background characteristics, prior general confidence in vaccine safety, prior confidence in the vaccine licensing authority, and prior vaccine hesitancy.

Figure A3 in Section SA2 of the supplemental appendix presents the means of “COVID arm” symptoms predicted by background characteristics, prior general confidence in vaccine safety, prior confidence in the vaccine licensing authority, and prior vaccine hesitancy $\varvec{X}_{i}$ for two groups subsampled by whether they experienced the symptoms. Respondents who experienced the symptoms are shown to have a higher predicted median probability of the symptoms. Additionally, Fig. A3 also shows that the mean of either subsample approaches neither 1 nor 0. This allowed us to balance the sample by weighting $\varvec{X}_{i}$.

Our estimates of $\tau \left( \varvec{x}\right)$ in Eq. (5) deployed augmented inverse propensity weighting (AIPW) to estimate $E\left[ \tau \left( \varvec{x}\right) \right]$. We also adopted a conservative approach for estimating the confidence intervals, as suggested by Holm [52].

## Results

### Confidence in vaccine safety

**Fig. 1:**
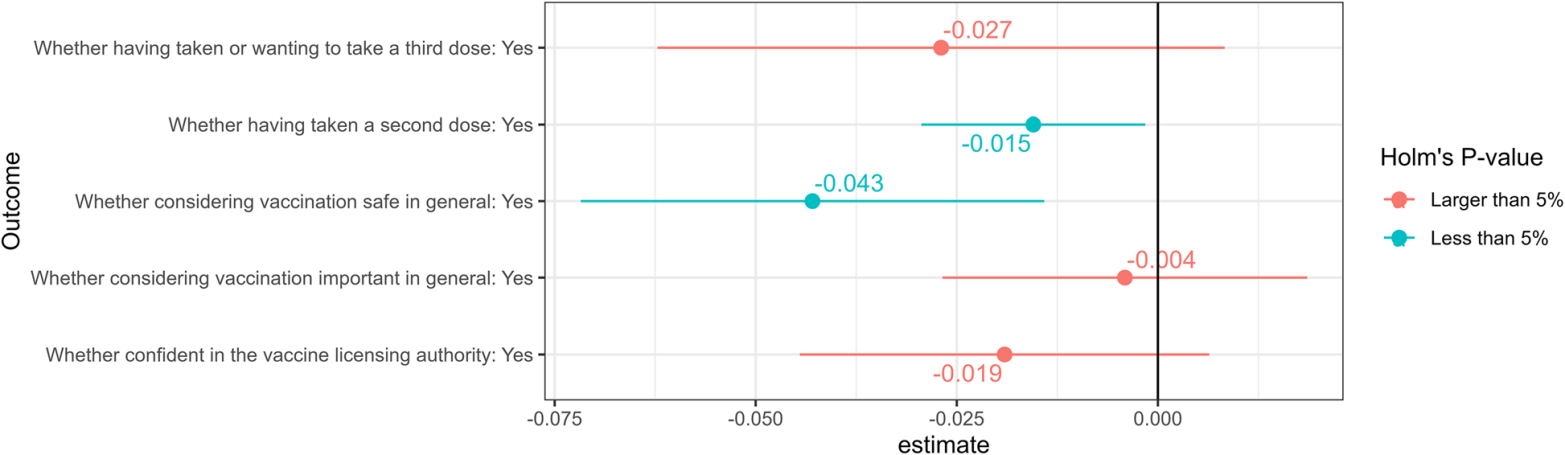
Impact of “COVID arm” symptoms on confidence in vaccine safety. *Notes:* According to Eqs.  (2) and (5), the estimates indicate the change in the predicted probability of taking a second dose and taking or wanting to take a third dose. The confidence interval was adjusted by the Bonferroni method, in addition to the *p*-values adjusted by the Holm method [52]. Point estimates of confidence intervals are presented in Table A3 in Section SA3 of the supplemental appendix

Figure 1 presents the estimated average treatment effects $E\left[ \tau \left( \varvec{x}\right) \right]$ with the sampling weight characterized by Eq. (5). The scale of the horizontal axis is the predicted change due to experiencing “COVID arm” symptoms in terms of the probability of having taken or wanting to take a third dose ($Y_{v^{5}, i}$ in Eq. (2)); having taken a second dose ($Y_{v^{4}, i}$); considering vaccination in general to be safe ($Y_{v^{1}, i}$); acknowledging the importance of vaccination ($Y_{v^{3}, i}$); and having confidence in the vaccine licensing authority ($Y_{v^{2}, i}$).

Figure 1 demonstrates that experiencing “COVID arm” symptoms significantly decreased confidence in the safety of vaccines by 4.3 percentage points. Since the sample mean of prior confidence in vaccine safety of respondents who happened to experience “COVID arm” symptoms in the first wave was 68%, as presented in Table A1 in Section SA1 of the supplemental appendix, the adverse impact was approximately 6.3% of the sample mean. Additionally, experiencing “COVID arm” symptoms significantly reduced the probability of taking a second dose of COVID-19 vaccines by 1.5 percentage points. The reduction in the probability of taking the next dose indicates a rise in vaccine hesitancy.

However, “COVID arm” symptoms only insignificantly negatively affected confidence in the vaccine licensing authority. Furthermore, “COVID arm” symptoms barely affected the general acknowledgment of the importance of vaccination. Belief updates about specific COVID-19 vaccines due to experiencing “COVID arm” significantly lowered the willingness to take a next dose of these specific vaccines but did not significantly affect general acknowledgment of the importance of vaccination and general confidence in the vaccine licensing authority.

In summary, downward belief updates about vaccine side effects due to experiencing “COVID arm” symptoms significantly adversely affected participants’ confidence in the safety of vaccination and their probability of taking a second dose. However, the belief updates did not affect the acknowledged importance of vaccination and only insignificantly affected general confidence in the vaccine licensing authority. We interpreted the latter insignificant results as indicating that the recognition of the vaccination’s importance and the general confidence in the vaccine licensing authority already factored in the uncertainty regarding vaccination, and hence, the acknowledgment of the vaccinations’ importance and the confidence in the vaccine licensing authority were tolerant to belief updates regarding the now estimable risks of side effects of these specific vaccines.

Figures A5 and A6 in Section SA5 of the supplemental appendix present the approximated conditional average treatment effects characterized by Eq. (5) on the confidence in vaccine safety ($Y_{v^{1}, i}$) and the probability of taking a second dose and of taking or wanting to take a third dose ($Y_{v^{4}, i}$, $Y_{v^{5}, i}$), which captures vaccine hesitancy, by linearly regressing the AIPW score function of the treatment effects on the background characteristics, prior general confidence in vaccine safety, prior confidence in the vaccine licensing authority, and prior vaccine hesitancy $\varvec{X}_{i}$. No element had a significant association with the treatment effect of “COVID arm” symptoms. This finding means that associations between the treatment effects and background characteristics, prior general confidence in vaccine safety, prior confidence in the vaccine licensing authority, and prior vaccine hesitancy were, if present, weak. The adverse impacts of “COVID arm” symptoms depended on belief updates regarding the side effects of vaccines across those who were and were not confident in vaccination before experiencing “COVID arm” symptoms.

### Confidence in science

**Fig. 2:**
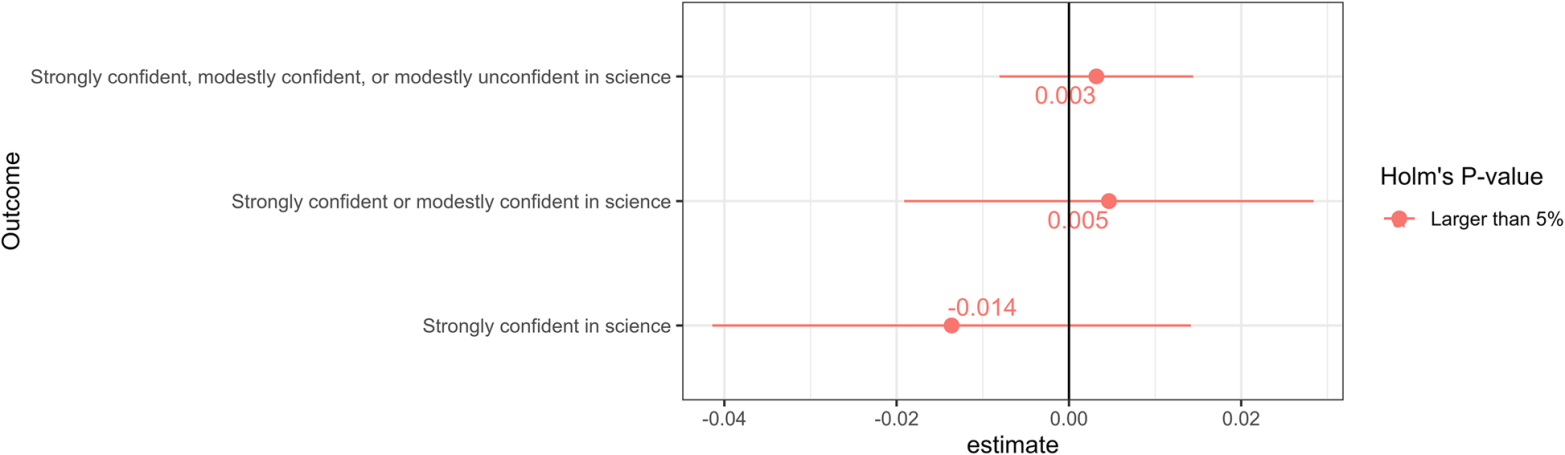
Impacts of “COVID arm” symptoms on confidence in science. *Notes:* Point estimates of confidence intervals are presented in Table A4 of Section SA3 of the supplemental appendix

In the second survey wave conducted in March 2022, we also asked the respondents about their confidence in science in general, as described by Eq. (3), as a supplementary study. Figure 2 presents $E\left[ \tau \left( \varvec{x}\right) \right]$ on confidence in science in general, characterized by Eq. (5). The impact of “COVID arm” symptoms on one’s confidence in science in general was weak. Only the probability of being “strongly confident in science,” in Eq. (3), was insignificantly lowered by experiencing “COVID arm” symptoms.

We interpreted this result in parallel to the insignificant results for the acknowledged importance of vaccination and confidence in the vaccine licensing authority in Fig. 1. That is, we understand that confidence in science in general already factored in the chance of uncertain outcomes following vaccination. Therefore, confidence in science in general was tolerant of belief updates, specifically in terms of beliefs about COVID-19 vaccines.

Additionally, Fig. A7 in Section SA5 of the supplemental appendix presents the approximated conditional average treatment effects on general confidence in science characterized by Eq. (5) for Eq. (4) by linearly regressing the AIPW score function on background characteristics, prior general confidence in vaccine safety, prior confidence in the vaccine licensing authority, and prior vaccine hesitancy $\varvec{X}_{i}$. The results show the insignificant effect of experiencing “COVID arm” symptoms on confidence in science in general across background characteristics, prior general confidence in vaccine safety, prior confidence in the vaccine licensing authority, and prior vaccine hesitancy.

## Discussion

Schmelz and Bowles found that better knowledge about vaccine efficacy and trust in public institutions were associated with higher support for a policy of mandated vaccination [28]. However, they did not identify what determines beliefs about vaccines in the form of knowledge. Algan et al. and Eichengreen et al. found general associations between trust in science and scientists and protective behaviors [35, 36]. Like Schmelz and Bowles, they did not address beliefs about vaccines and protective behaviors that determine trust in vaccines and protective behaviors, either.

In contrast, we used “COVID arm” as an exogenous shock that could affect beliefs about vaccine side effects. Then, we investigated whether such belief updates affected confidence in vaccine safety, confidence in the vaccine licensing authority, and vaccine hesitancy. Our contribution is that we identify a causal relationship between an unexpected and uncomfortable experience with novel vaccines, through belief updates about the side effects of vaccines, and a rise in vaccine hesitancy. We measured the effects of belief updates of the same individuals in the same country, among both those who were already generally confident in vaccination and those who were already generally skeptical.

We found negative impacts of downward belief updates about COVID-19 vaccine side effects on confidence in vaccine safety and the willingness to take the next dose of specific vaccines. However, we did not find a significant change in the acknowledgment of the general importance of vaccination, general confidence in the vaccine licensing authority, and confidence in science in general.

## Conclusion

In February 2021 and March 2022, we asked the same respondents about their confidence in vaccine safety and confidence in the vaccine licensing authority, and in March 2022, we asked whether they had experienced “COVID arm” symptoms if they had been vaccinated. We found that experiencing “COVID arm” symptoms significantly decreased their confidence in the safety of vaccination and the probability of taking a second dose of COVID-19 vaccines. Adverse impacts were found regardless of the levels of prior general confidence in vaccine safety, prior confidence in the vaccine licensing authority, and prior vaccine hesitancy. Chen found that not only the final coverage rate but also the speed of vaccination spread significantly affect the infection and mortality rates [53]. The finding extends the policy implications of our results that “COVID arm” at least delayed the timing of taking the next dose as of our survey.

Meanwhile, experiencing “COVID arm” symptoms did not significantly affect the acknowledgment of the general importance of vaccination, general confidence in the vaccine licensing authority, or general confidence in science. We interpret the irrelevance of these results as being because these acknowledgments already factored in possible uncertainties regarding specific vaccines. Downward belief updates about specific vaccines did not significantly alter the general recognition of the importance of vaccination, confidence in the vaccine licensing authority, or confidence in science in general.

It has been predicted that a higher risk of adverse side effects is associated with a higher probability of avoiding the vaccine [11–25]. As a further step to be added to these results, we investigated how a change in beliefs about the risk of side effects affects take-up of the next dose and confidence in vaccine safety. In other words, we studied how people’s beliefs about unknown treatment are formed and how they could change as they acquire their own experiences. Our results show that an update of knowledge about vaccines through side effects affected take-up of the next dose and confidence in vaccine safety.

Furthermore, Hosogaya et al. found significant relationships between acceptance of hypothetical oral antiviral drugs for COVID-19 treatment and beliefs about the treatment [54]. Given the results, the impacts of belief updates about a new treatment could be generalized beyond vaccination.

Dynamic evolution of peoples’ beliefs is associated with dynamic change in their confidence in and willingness to take a medical treatment. Our results suggest that negative events such as adverse side effects deliver downward updates of beliefs about the medical treatment. Nonetheless, unless adverse side effects are severe, the downward update of the belief about the side effects did not significantly affect deeply rooted general confidence in public institutions such as the vaccine licensing authority and science.

Obviously, recipients’ own experience of vaccination is not the sole opportunity for belief updates. Tomioka et al. presented that a higher density of public servants and physicians per population was associated with higher rates of vaccination among the elderly population [55]. Although they did not discuss a possible channel of the effects, opportunities to gain correct knowledge about a medical treatment might upwardly affect prior beliefs about a new medical treatment. If this holds, more frequently touching correct knowledge might mitigate the downward updates of beliefs due to experiencing adverse side effects.

While our focus was on potentially different updates of beliefs about COVID-19 vaccines and their impacts on confidence in the safety of COVID-19 vaccines, another important issue is the possible divide between vaccine recipients and those who are vaccine hesitant. As presented in Table A1 in the supplemental appendix, confidence in the safety of COVID-19 vaccines substantially fell among respondents who never received COVID-19 vaccines from February 2021 to March 2022. Although vaccine acceptance rates have risen over time, there remain entrenched vaccine-hesitant individuals who are concerned about adverse side effects and distrust vaccine safety in both the US and Japan [22, 56]. The entrenched vaccine hesitancy of those who never received a dose of COVID-19 vaccines is another issue for policy making.

One limitation of our research is that we did not address the possible effects of further belief evolution about the side effects of the vaccines. If people who were better informed of the COVID-19 vaccines’ side effects were less vaccine hesitant [32], taking more doses would have reduced vaccine hesitancy through the acquisition of updated information and experiences regarding the effects of the vaccines. This inference is consistent with our observation presented in Table A1 in Section SA1 of the supplemental appendix. In our sample, recipients of the first dose of COVID-19 vaccines became more confident in vaccine safety, while those who did not take the first dose became less confident.

However, second doses of either the Moderna (mRNA-1273) or Pfizer (BNT162b2) vaccine were more likely to be accompanied by side effects than the first doses [57, 58]. If this higher likelihood of the second dose’s side effects was unexpected, it would have negatively affected the willingness to take a third dose. These forces of acquisition of new information and a possibly unexpected higher likelihood of side effects of the second dose could work in opposite directions regarding the willingness to take third and fourth doses. It is left to our next study to identify which force was dominant [59].

### Supplementary Information

**Additional file 1.** Supplemental appendix.

## Data Availability

The datasets generated by the survey research and analyzed during the current study and the original questionnaire of the survey will be publicly available after cleaning at the Center for Social Research and Data Archives, Institute of Social Science, The University of Tokyo (https://csrda.iss.u-tokyo.ac.jp/english/). Until they become publicly available at the institution, they will be available from the corresponding author, Masaki Nakabayashi, upon request. An English translation of the questionnaire of the survey is provided in section SA6 of the supplemental appendix.

## Footnotes

1 Detailed information about Rakuten Insight’s respondent pool is available from its website. https://insight.rakuten.co.jp/download/PanelProfile_EN.pdf and https://insight.rakuten.co.jp/download/PanelCharacteristicSurveyEN.pdf.

2 The results of the census are available from Statistic Bureau of Japan, Government of Japan: https://www.stat.go.jp/english/data/kokusei/index.html Accessed on March 20, 2023.

3 https://info.vrs.digital.go.jp/ Accessed on July 16, 2022.
