# Supplementary material for: Updated beliefs and shaken confidence: evidence from vaccine hesitancy caused by experiencing “COVID arm”
Source: BMC Infect Dis. 2023 Sep 18;23:612. doi: 10.1186/s12879-023-08558-5 (PMC10507958; doi:10.1186/s12879-023-08558-5)
Supplement: Supplementary file 1 — Additional file 1. Supplemental appendix. [file 12879_2023_8558_MOESM1_ESM.pdf]

Supplemental appendix

SA 1 Descriptive statistics

Table A1 presents descriptive statistics of the respondents' background characteristics, general confidence in vaccine safety, confidence in the vaccine licensing authority and vaccine hesitancy asked about in both waves and confidence in science in general asked about in the second wave. We first classified the sample into respondents who took a first dose of COVID-19 vaccines and those who did not. Next, we divided the respondents who took a first dose into those who experienced "COVID arm" symptoms and those who did not.

We found differences between those who had taken a first dose as of March 2022 and those who had not in terms of income levels, age, education level, and political beliefs. On average, compared to vaccine recipients, vaccine-hesitant individuals were younger and less educated, more likely to be unmarried, and more likely to be politically independent. A striking difference between the vaccine recipients and those who were vaccine hesitant was in the evolution of their confidence in vaccine safety. Among those who had taken the first dose of the COVID-19 vaccine as of March 2022, 25% were not confident in vaccine safety in February 2021, but the percentage fell to 18% in March 2022. The experience of taking the COVID-19 vaccine was accompanied by a rise in confidence in vaccine safety. In contrast, among those who had not taken the first dose of COVID-19 vaccine as of March 2022, 56% were not confident in vaccine safety in February 2021, and the percentage climbed to 70% in March 2022. Thus, the division between vaccine recipients and vaccine-hesitant individuals in terms of confidence in vaccine safety deepened. However, the deepening of this division, which already existed in Japan before the COVID-19 vaccine rollout, is not the focus of our work. Our interest is in how "COVID arm" symptoms, as an unexpected shock, affected beliefs about vaccination among recipients of the first dose of the COVID-19 vaccine. Therefore, we used the 8,321 respondents who received a first dose and participated in both the first and second waves as our primary sample.

Out of the 8,321 respondents, 30 in total did not respond to the question about employment status. The information was necessary to weight our sample in terms of sex, age, and employment status pursuant to the composition in the latest population census. We thus primarily analyzed the remaining 8,291 respondents.

Table A1. Descriptive statistics.

Characteristics	Have taken first dose of COVID-19 vaccine		Have not taken first dose of COVID-19 vaccine
	COVID arm: Yes N = 3,214[1]	COVID arm: No N = 5,098[1]	N = 1,356[1]
**Confident in science, Mar 2022**			
Strongly confident	585 (18%)	1,056 (21%)	160 (12%)
Confident to some extent	2,248 (70%)	3,465 (68%)	869 (64%)
Not very confident	327 (10%)	489 (9.6%)	227 (17%)
Other[2]	54 (1.7%)	88 (1.7%)	100 (7.4%)
**Confident in vaccine safety in general, Mar 2022**			
Confident	2,477 (77%)	4,160 (82%)	384 (28%)
Not confident	715 (22%)	909 (18%)	954 (70%)
Other[2]	22 (0.7%)	29 (0.6%)	18 (1.3%)
**Thinking about vaccination important in general, Mar 2022**			
Important	2,854 (89%)	4,551 (89%)	531 (39%)
Not important	286 (8.9%)	440 (8.6%)	786 (58%)
Other[2]	74 (2.3%)	107 (2.1%)	39 (2.9%)
**Confident in vaccine licensing by the Ministry of Health, Labour and Welfare, Mar 2022**			
Confident	2,631 (82%)	4,271 (84%)	409 (30%)
Not confident	553 (17%)	790 (15%)	919 (68%)
Other[2]	30 (0.9%)	37 (0.7%)	28 (2.1%)
**Have taken first dose of COVID-19 vaccine, Mar 2022**			
Yes	3,214 (100%)	5,098 (100%)	0 (0%)
No	0 (0%)	0 (0%)	1,277 (94%)
Other[2]	0 (0%)	0 (0%)	79 (5.8%)
**Have taken second dose of COVID-19 vaccine, Mar 2022**			
Yes	3,113 (97%)	5,008 (98%)	0 (0%)
Do not remember	56 (1.7%)	57 (1.1%)	1,356 (100%)
Other[2]	45 (1.4%)	33 (0.6%)	0 (0%)
**Have taken third dose of COVID-19 vaccine, Mar 2022**			
Yes	1,174 (37%)	1,939 (38%)	0 (0%)
No	1,911 (59%)	3,028 (59%)	0 (0%)
Do not remember	129 (4.0%)	131 (2.6%)	1,356 (100%)
**Will take third dose of COVID-19 vaccine, Mar 2022**			
Will take	1,453 (45%)	2,427 (48%)	0 (0%)
Have not decided	345 (11%)	462 (9.1%)	0 (0%)
Do not know	1,306 (41%)	2,076 (41%)	1,356 (100%)
Other[2]	110 (3.4%)	133 (2.6%)	0 (0%)
**Type of COVID-19 vaccine as of Mar 2022**			
Pfizer (BNT162b2)	2,201 (68%)	4,038 (79%)	0 (0%)
Moderna (mRNA-1273)	922 (29%)	895 (18%)	0 (0%)
Do not remember	44 (1.4%)	66 (1.3%)	1,356 (100%)
Other[2]	47 (1.5%)	99 (1.9%)	0 (0%)
**Have contracted COVID-19, Feb 2021**			
No	3,116 (97%)	4,943 (97%)	1,257 (93%)
Other[2]	98 (3.0%)	155 (3.0%)	99 (7.3%)
**Have taken any vaccines, Feb 2021**			
Yes	1,624 (51%)	2,532 (50%)	443 (33%)
No	1,550 (48%)	2,497 (49%)	860 (63%)
Other[2]	40 (1.2%)	69 (1.4%)	53 (3.9%)
**Confident in vaccine safety in general, Feb 2021**			
Trust	2,189 (68%)	3,577 (70%)	484 (36%)
Do not trust	869 (27%)	1,269 (25%)	753 (56%)
Do not know	156 (4.9%)	252 (4.9%)	119 (8.8%)
**Consider vaccination important, Feb 2021**			
Important	2,693 (84%)	4,269 (84%)	709 (52%)
Not important	372 (12%)	594 (12%)	506 (37%)
Do not know	149 (4.6%)	235 (4.6%)	141 (10%)
**Trust in vaccine licensing by the Ministry of Health, Labour and Welfare Feb 2021**			
Trust	2,363 (74%)	3,803 (75%)	557 (41%)
Do not trust	603 (19%)	929 (18%)	604 (45%)
Other[2]	248 (7.7%)	366 (7.2%)	195 (14%)
**Have avoided vaccination due to concerns about the risk or efficacy of**			

**vaccines, Feb 2021.**			
Yes	569 (18%)	776 (15%)	429 (32%)
No	2,536 (79%)	4,146 (81%)	827 (61%)
Other[2]	109 (3.4%)	176 (3.5%)	100 (7.4%)
**Have postponed vaccination recommended by a doctor, Feb 2021**			
Yes	264 (8.2%)	381 (7.5%)	192 (14%)
No	2,869 (89%)	4,581 (90%)	1,072 (79%)
Other[2]	81 (2.5%)	136 (2.7%)	92 (6.8%)
**Trust whose opinions regarding vaccination, Feb 2021**			
Family or good friends	215 (6.7%)	311 (6.1%)	147 (11%)
Doctor	2,332 (73%)	3,798 (74%)	678 (50%)
Internet	309 (9.6%)	425 (8.3%)	227 (17%)
Other sources	284 (8.8%)	423 (8.3%)	216 (16%)
Other[2]	74 (2.3%)	141 (2.8%)	88 (6.5%)
**Think COVID-19 vaccination should be mandatory, Feb 2021**			
Yes	1,591 (50%)	2,671 (52%)	295 (22%)
No	1,446 (45%)	2,152 (42%)	931 (69%)
Do not want to answer	157 (4.9%)	255 (5.0%)	123 (9.1%)
Other[2]	20 (0.6%)	20 (0.4%)	7 (0.5%)
**Think COVID-19 vaccines should be fully subsidized by the government, Feb 2021**			
Yes	2,730 (85%)	4,393 (86%)	965 (71%)
No	373 (12%)	557 (11%)	281 (21%)
Other[2]	111 (3.5%)	148 (2.9%)	110 (8.1%)
**Gender**			
Male	1,555 (48%)	2,955 (58%)	675 (50%)
Female	1,659 (52%)	2,143 (42%)	681 (50%)
Other[2]	0 (0%)	0 (0%)	0 (0%)
**Age, Feb 2021**	49 (39, 59)	51 (42, 61)	45 (35, 53)
**Prefecture of residence, Feb 2021**			
Saitama	184 (5.7%)	278 (5.5%)	71 (5.2%)
Chiba	171 (5.3%)	272 (5.3%)	66 (4.9%)
Tokyo	470 (15%)	764 (15%)	176 (13%)
Kanagawa	297 (9.2%)	437 (8.6%)	114 (8.4%)
Aichi	206 (6.4%)	321 (6.3%)	92 (6.8%)
Osaka	245 (7.6%)	387 (7.6%)	134 (9.9%)
Other[2]	1,641 (51%)	2,639 (52%)	703 (52%)
**Marital status, Feb 2021**			
Unmarried	858 (27%)	1,252 (25%)	570 (42%)
Married	2,067 (64%)	3,404 (67%)	660 (49%)
Divorced or widowed	277 (8.6%)	417 (8.2%)	120 (8.8%)
Other[2]	12 (0.4%)	25 (0.5%)	6 (0.4%)
**Number of children, Feb 2021**			
0	1,329 (41%)	2,013 (40%)	773 (57%)
1.	538 (17%)	814 (16%)	197 (15%)
2.	988 (31%)	1,638 (32%)	257 (19%)
3.	322 (10%)	536 (11%)	98 (7.3%)
4.	23 (0.7%)	73 (1.4%)	20 (1.5%)
5. or more	9 (0.3%)	18 (0.4%)	5 (0.4%)
Other[2]	5	6	6
**Working status, Feb 2021**			
Employed	2,311 (72%)	3,703 (73%)	946 (70%)
Unemployed	894 (28%)	1,384 (27%)	408 (30%)
Other[2]	9 (0.3%)	11 (0.2%)	2 (0.1%)
**Employment type, Feb 2021**			
Nonregular worker	625 (19%)	1,010 (20%)	259 (19%)
Regular worker	1,474 (46%)	2,315 (45%)	524 (39%)
Self-employed	180 (5.6%)	337 (6.6%)	138 (10%)
Do not know	906 (28%)	1,399 (27%)	412 (30%)
Other[2]	29 (0.9%)	37 (0.7%)	23 (1.7%)
**Job title, Feb 2021**			
No title	1,359 (70%)	2,045 (67%)	571 (80%)
Team leader	86 (4.4%)	120 (3.9%)	24 (3.3%)
Assistant manager	197 (10%)	307 (10%)	59 (8.2%)
Manager	191 (9.9%)	364 (12%)	39 (5.4%)
Division manager	102 (5.3%)	221 (7.2%)	25 (3.5%)
No response[3]	1,279	2,041	638
**Employer size, Feb 2021**			
1–4 employees	84 (4.4%)	119 (4.0%)	44 (6.2%)
5–29 employees	294 (16%)	481 (16%)	133 (19%)
30–99 employees	315 (17%)	486 (16%)	139 (19%)
100–499 employees	432 (23%)	695 (23%)	153 (21%)
500 or more	765 (40%)	1,222 (41%)	246 (34%)
Public servant	143 (7.0%)	216 (6.7%)	44 (5.8%)
No response[3]	1,324	2,095	641
No response[3]	1,181	1,879	597

**Personal income, Feb 2021**			
Less than 0.50 million yen	527 (16%)	681 (13%)	316 (23%)
0.50–0.99 million yen	263 (8.2%)	344 (6.8%)	118 (8.7%)
1.00–1.49 million yen	249 (7.8%)	369 (7.3%)	97 (7.2%)
1.50–1.99 million yen	161 (5.0%)	306 (6.0%)	93 (6.9%)
2.0–2.49 million yen	234 (7.3%)	393 (7.7%)	100 (7.4%)
2.50–2.99 million yen	188 (5.9%)	318 (6.3%)	93 (6.9%)
3.00–3.99 million yen	359 (11%)	610 (12%)	138 (10%)
4.00–4.99 million yen	340 (11%)	563 (11%)	133 (9.8%)
5.00 million yen or more	882 (28%)	1,496 (29%)	263 (19%)
No response[3]	11	18	5
**Household income, Feb 2021**			
2.00–2.49 million yen	159 (4.9%)	250 (4.9%)	80 (5.9%)
2.50–2.99 million yen	160 (5.0%)	251 (4.9%)	85 (6.3%)
3.00–3.99 million yen	355 (11%)	611 (12%)	164 (12%)
4.00–4.99 million yen	386 (12%)	640 (13%)	156 (12%)
5.00–5.99 million yen	377 (12%)	579 (11%)	143 (11%)
6.00–6.99 million yen	299 (9.3%)	453 (8.9%)	115 (8.5%)
7.00–7.99 million yen	291 (9.1%)	444 (8.7%)	113 (8.3%)
8.00–8.99 million yen	222 (6.9%)	371 (7.3%)	66 (4.9%)
9.00–9.99 million yen	191 (5.9%)	271 (5.3%)	48 (3.5%)
10 million yen or more	496 (15%)	781 (15%)	162 (12%)
Other	278 (8.6%)	447 (8.8%)	224 (17%)
**Highest degree, Feb 2021**			
Junior high school	36 (1.1%)	51 (1.0%)	46 (3.4%)
High school	705 (22%)	1,186 (23%)	375 (28%)
Some college	722 (23%)	1,026 (20%)	339 (25%)
College	1,516 (47%)	2,501 (49%)	541 (40%)
Graduate school	228 (7.1%)	324 (6.4%)	53 (3.9%)
Other[2]	7	10	2
**Party support, Feb 2021**			
Independent	1,769 (55%)	2,762 (54%)	864 (64%)
Opposition parties	650 (20%)	978 (19%)	242 (18%)
Ruling parties	787 (24%)	1,353 (27%)	245 (18%)
Other[2]	8 (0.2%)	5 (<0.1%)	5 (0.4%)
**Satisfaction with current politics, Feb 2021**			
Substantially satisfied	28 (0.9%)	66 (1.3%)	25 (1.9%)
Satisfied to some extent	338 (11%)	589 (12%)	95 (7.0%)
Neither satisfied nor dissatisfied	811 (25%)	1,313 (26%)	353 (26%)
Dissatisfied to some extent	903 (28%)	1,346 (26%)	293 (22%)
Substantially dissatisfied	1,125 (35%)	1,782 (35%)	585 (43%)
Other[2]	9	2	5
**Self-perceived degree of right-leaning political beliefs 0: Completely left; 10: Completely right**			
	6.00 (6.00, 7.00)	6.00 (6.00, 7.00)	6.00 (6.00, 6.00)
other[2]	57	99	43
**Self-perceived social status 1: Highest; 10: Lowest**			
	6.00 (5.00, 8.00)	6.00 (5.00, 8.00)	6.00 (6.00, 8.00)
other[2]	37	61	35

[1] Number of responses (%) or median (interquartile range).
[2] Responses whose shares are 5% or less are summed and collectively referred to as "other". They mostly arose due to respondents skipping the question.
[3] This question was irrelevant to the respondent or the respondent skipped the question. We allowed respondents to skip a question if they wanted to.

SA 2 Balancing covariates

Figure A1 presents standardized mean differences in the probability of experiencing "COVID arm" symptoms between unadjusted and adjusted groups regarding four primary background characteristics: age, sex, Moderna (mRNA-1273) vaccine recipient status, and chronic disease status.

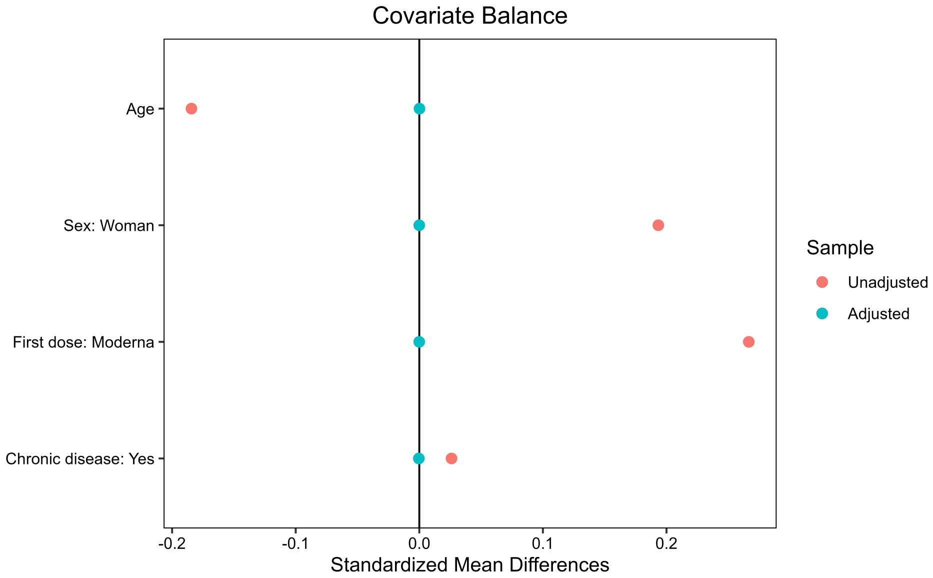

Fig. A1: Covariate balances for primary variables.

Figure A2 presents linear regressions of whether respondents experienced "COVID arm" symptoms on background characteristics, prior general confidence in vaccine safety, prior confidence in the vaccine licensing authority, and prior vaccine hesitancy. Even after the sample was balanced for the four primary background characteristics presented in Fig. A1, the probability of experiencing "COVID arm" symptoms was associated with a wide range of other background characteristics, prior general confidence in vaccine safety, prior confidence in the vaccine licensing authority, and prior vaccine hesitancy, although the absolute values of the coefficients were smaller.

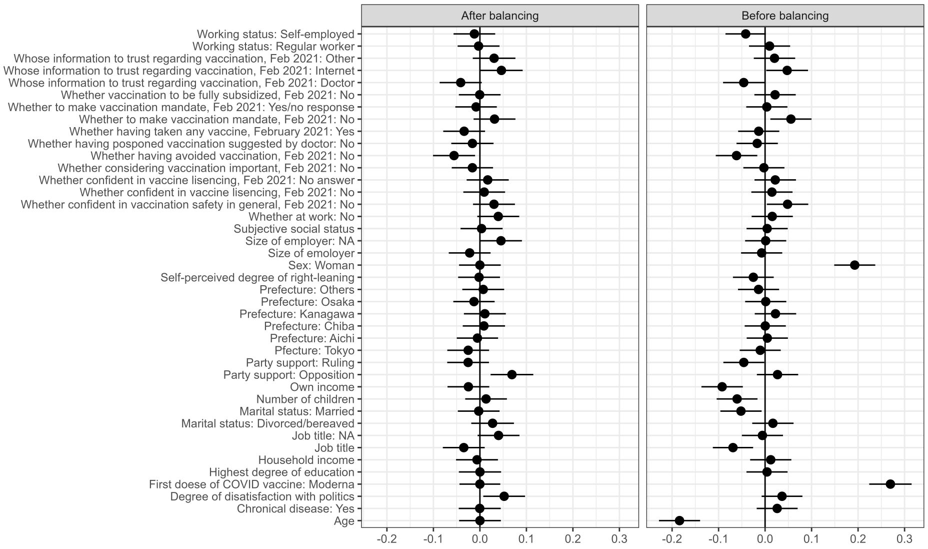

Fig. A2: Covariate balances for all background characteristics and prior beliefs.

Table A2 presents the degrees of importance of characteristics to calculate the propensity scores. The importance indicates the frequency with which the variable was used by the random forest algorithm to estimate the propensity score [47]. While having taken the Moderna vaccine is by far the most important characteristic, other characteristics, such as prior confidence in vaccine safety as of February 2021, prior confidence in the vaccine licensing authority, prior acknowledgment of the importance of vaccination, and prior vaccine hesitancy captured by experiences of postponing or avoiding vaccination, are also modestly important. Therefore, we condition all the characteristics when calculating mean functions and use the inverses of propensity scores to adjust differences in the predicted occurrence of "COVID arm" symptoms.

Table A2. Importance of characteristics.

Characteristics	Importance
First dose of COVID vaccine: Moderna	0.4536
Age	0.1489
Sex: Female	0.1239
Individual income	0.0661
Household income	0.0238
Subjective social status	0.0203
Self-perceived degree of right-leaning	0.0123
Whose information regarding vaccination is trusted, Feb 2021: Internet	0.0120
Marital status: Married	0.0111
Has avoided vaccination, Feb 2021: No	0.0104
Degree of dissatisfaction with politics	0.0081
Employment status: No	0.0080
Size of employer: NA	0.0066
Number of children	0.0063
Highest educational degree	0.0056
Job title	0.0054
Prefecture: Osaka	0.0053
Has postponed vaccination suggested by doctor: No	0.0050
Whose information regarding vaccination is trusted, Feb 2021: Doctor	0.0048
Agrees that vaccination should be fully subsidized, Feb 2021: No	0.0048
Confident in vaccine safety in general, Feb 2021: No	0.0046
Agrees with a vaccination mandate, Feb 2021: No	0.0045
Chronic disease: Yes	0.0043
Size of employer	0.0039
Job title: NA	0.0038
Party support: Opposition	0.0037
Has taken any vaccine, Feb 2021: Yes	0.0035
Prefecture: Tokyo	0.0033
Considers vaccination important, Feb 2021: No	0.0030
Whose information regarding vaccination is trusted, Feb 2021: Other	0.0028
Confident in vaccine licensing, Feb 2021: No answer	0.0027
Working status: Regular worker	0.0025
Prefecture: Kanagawa	0.0023
Confident in vaccine licensing, Feb 2021: No	0.0022
Party support: Ruling	0.0021
Prefecture: Others	0.0019
Agrees with a vaccination mandate, Feb 2021: Yes/no response	0.0018
Prefecture: Aichi	0.0014
Marital status: Divorced/widowed	0.0013
Prefecture: Chiba	0.0010
Working status: Self-employed	0.0010

Let us divide the sample into two subsamples depending on whether they actually experienced “COVID arm” symptoms and then predict the means of “COVID arm” symptoms by background characteristics, prior general confidence in vaccine safety, prior confidence in the vaccine licensing authority, and prior vaccine hesitancy. As Fig. A3 shows, respondents who experienced “COVID arm” symptoms had a higher predicted median probability of “COVID arm” symptoms conditional on background characteristics, prior general confidence in vaccine safety, prior confidence in the vaccine licensing authority, and prior vaccine hesitancy.

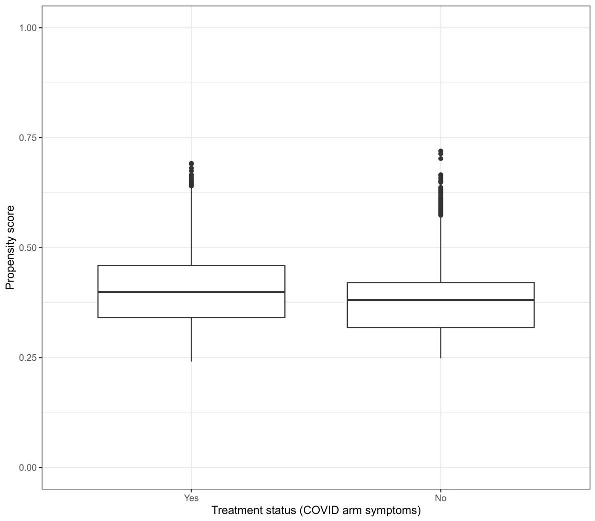

Fig. A3: Predicted probability of experiencing “COVID arm” symptoms conditional on background characteristics and prior beliefs.

SA 3 Point estimates of primary results

Table A3 presents point estimates of the results shown in Fig. 1.

Table A3. Point estimates of Fig. 1.

Outcome	Estimate	Std. error	Statistic	p value	Low confidence	High confidence	N
Considers vaccination safe in general: Yes	-0.04	0.01	-3.84	0.00	-0.07	-0.01	8291
Considers vaccination important in general: Yes	-0.00	0.01	-0.47	0.64	-0.03	0.02	8291
Confident in the vaccine licensing authority: Yes	-0.02	0.01	-1.93	0.05	-0.04	0.01	8291
Has taken a second dose: Yes	-0.02	0.01	-2.87	0.00	-0.03	-0.00	8291
Has taken or wants to take a third dose: Yes	-0.03	0.01	-1.97	0.05	-0.06	0.01	8291

Table A4 presents the point estimates of the results shown in Fig. 2.

Table A4. Point estimates of Fig. 2.

Outcome	Estimate	Std. error	Statistic	p value	Low confidence	High confidence	N
Strongly confident in science	-0.01	0.01	-1.17	0.24	-0.04	0.01	8291
Strongly confident or modestly confident in science	0.00	0.01	0.47	0.64	-0.02	0.03	8291
Strongly confident, modestly confident, or modestly unconfident in science	0.00	0.00	0.68	0.50	-0.01	0.01	8291

SA 4 Results without weighting by population census

Figure A4 presents the same treatment effects in Fig. 1 on all of 8,321 respondents who took the first dose of a COVID-19 vaccine and participated in both the first and second waves, without weighting the sample by sex, age, and employment status pursuant to the composition in the latest population census in 2020. The results did not differ qualitatively from those for the sample of 8,291 respondents weighted by the latest population census. Quantitatively, the balanced sample shows greater treatment effects on confidence in vaccine safety and willingness to take the second dose.

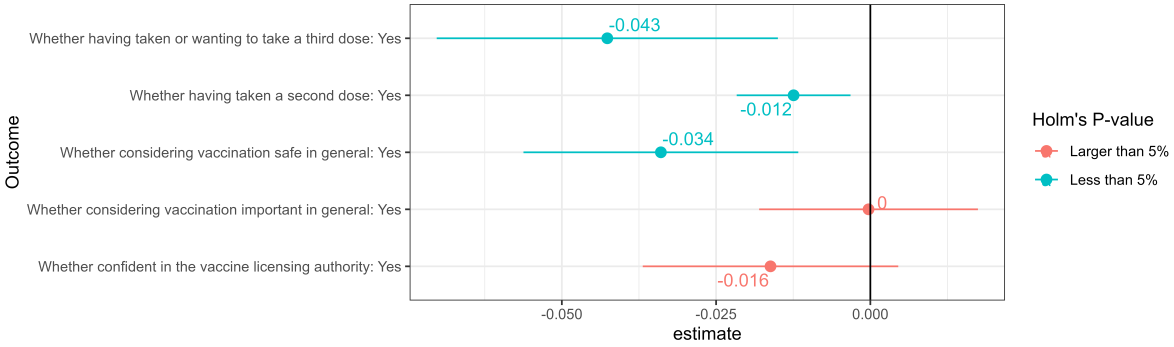

Fig. A4: Impact of "COVID arm" symptoms on confidence in vaccine safety: Without balancing by the population census.
Notes: According to Eqs. (2) and (5), the estimates indicate the change in the predicted probability of taking a second dose and taking or wanting to take a third dose. The confidence interval was adjusted by the Bonferroni method, in addition to the p-values adjusted by the Holm method [52].

SA 5 Supplementary results

Figure A5 presents a regression of the AIPW score function of treatment effects on the confidence in the vaccine licensing authority ($Y_{v^2,i}$ in Eq. (2)), acknowledgment of the importance of vaccination ($Y_{v^3,i}$), and general confidence in vaccine safety ($Y_{v^1,i}$), $\tau(\boldsymbol{x})$, on background characteristics, prior general confidence in vaccine safety, prior confidence in the vaccine licensing authority, and prior vaccine hesitancy $\boldsymbol{X}_i$.

These effects are estimated by the best linear projection, which regresses the average treatment effects on normalized $\boldsymbol{X}_i$ such that $E[\tau(\boldsymbol{x})|\boldsymbol{X}_i = \boldsymbol{x}] \approx \beta_0 + \boldsymbol{\beta} \times \boldsymbol{X}_i$, based on a debiased estimation method [45]. Thus, essentially, the best linear projection presents the treatment effects as a function of background characteristics, prior confidence in vaccine safety, prior confidence in the vaccine licensing authority, and prior vaccine hesitancy. Background characteristics, prior general confidence in vaccine safety, prior confidence in the vaccine licensing authority, and prior vaccine hesitancy were not significantly associated with the predicted treatment effects of "COVID arm" symptoms.

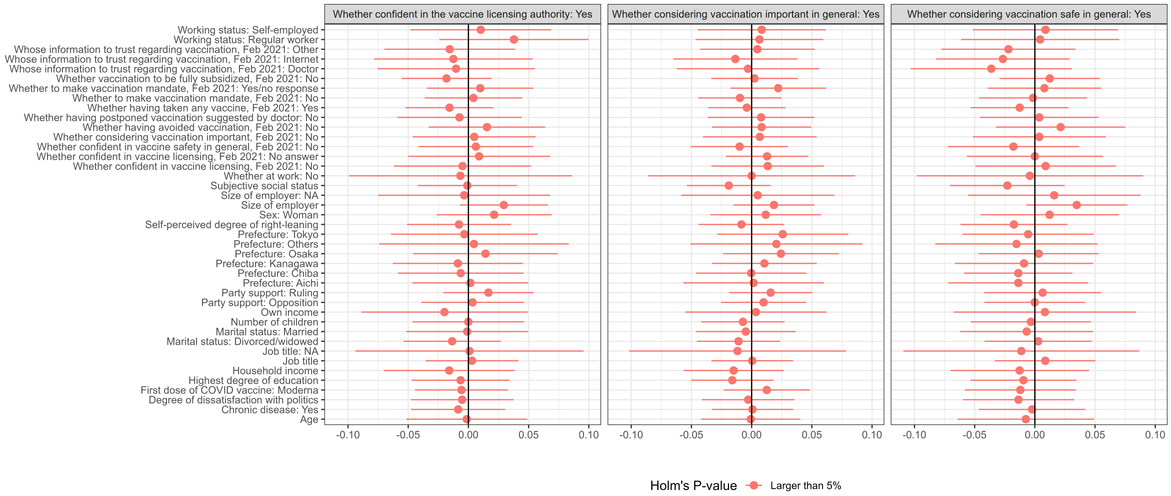

Fig. A5: Impacts of "COVID arm" symptoms on confidence in vaccine safety: Best linear projection.

Figure A6 presents a regression of the AIPW score function of the treatment effects on the probability of taking a second dose and the probability of taking ($Y_{v^4,i}$) taking or wanting to take a third dose ($Y_{v^5,i}$), which captures vaccine hesitancy estimated in the same way, $\tau(\boldsymbol{x})$, on background characteristics, prior confidence in vaccine safety, prior confidence in the vaccine licensing authority, prior acknowledgment of vaccination importance, and prior vaccine hesitancy $\boldsymbol{X}_i$. None of the background characteristics were significantly associated with the predicted treatment effects of "COVID arm" symptoms, nor were prior general confidence in vaccine safety, prior confidence in the vaccine licensing authority, or prior vaccine hesitancy.

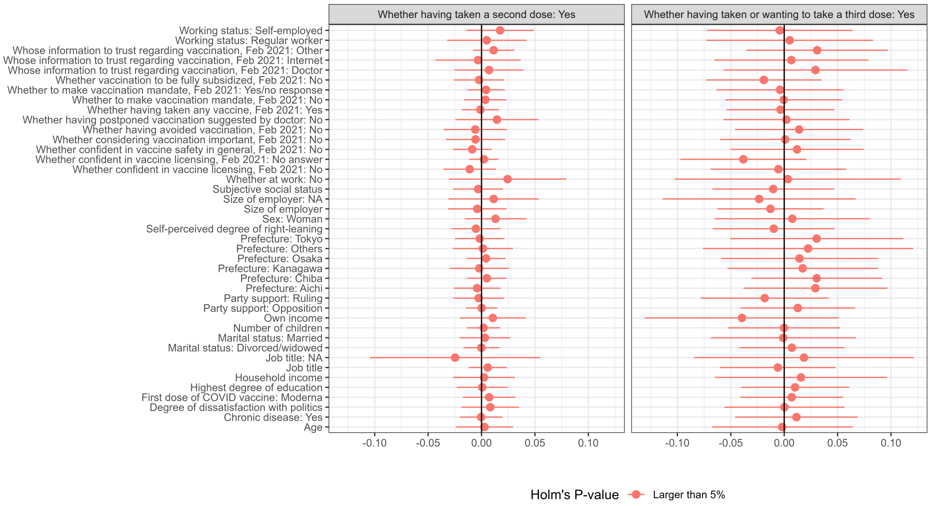

Fig. A6: Impacts of "COVID arm" symptoms on the probability of taking a second dose and of taking or wanting to take a third dose: Best linear projection.

Figure A7 presents a regression of the AIPW score function of the treatment effects on confidence in science in general ($Y_{s^1,i}$, $Y_{s^2,i}$, and $Y_{s^3,i}$ in Eq. (4)) on background characteristics, prior general confidence in vaccine safety, prior confidence in the vaccine licensing authority, and prior vaccine hesitancy $\boldsymbol{X}_i$. They are estimated by the best linear projection, $E[\tau(\boldsymbol{x})|\boldsymbol{X}_i = \boldsymbol{x}] \approx \beta_0 + \boldsymbol{\beta} \times \boldsymbol{X}_i$. The predicted treatment effects of "COVID arm" symptoms on confidence in science in general were not significantly associated with background characteristics, prior general confidence in vaccine safety, prior confidence in the vaccine licensing authority, or prior vaccine hesitancy $\boldsymbol{X}_i$.

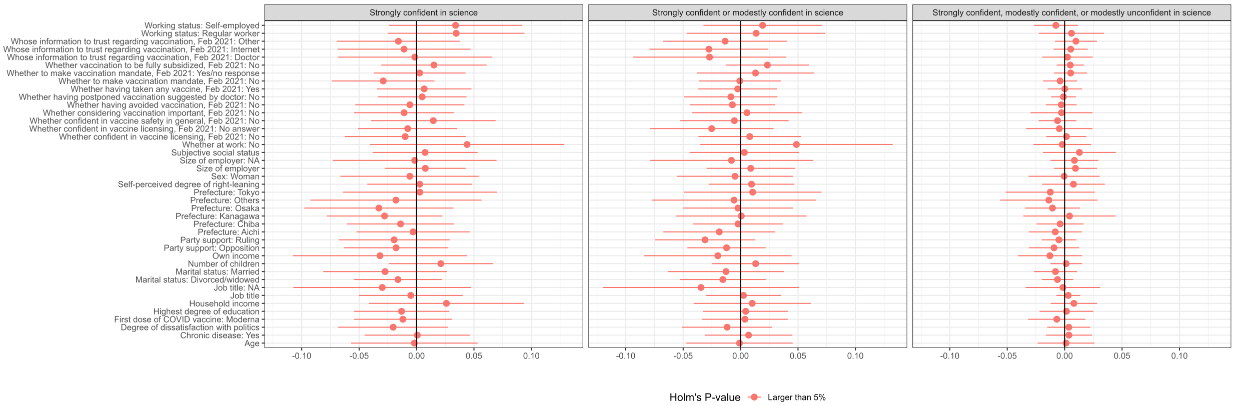

Fig. A7: Impacts of "COVID arm" symptoms on confidence in science: Best linear projection.

SA 6 Excerpt from the questionnaire

An English translation of an excerpt from the questionnaire in the first wave in February to Mach 2021 and the second wave in March 2022 is as follows.

1. Please tell us your gender. (Choose one)
    1. Male.
    2. Female.
2. How old are you?
3. Which prefecture do you live in? (Choose one)
4. In general, do you trust in science strongly, to some extent, not much, or not at all? (Choose one) [Asked only in the second wave in March 2022]
    1. Strongly trust.
    2. Moderately trust.
    3. Do not trust very much.
    4. Do not trust at all.
5. Do you trust scientists who belong to universities in this country and perform the tasks described below strongly, to some extent, not much, or not at all? (Choose one) [Asked only in the second wave in March 2022]
    1. Scientists working to serve the common good.
        1. Strongly trust.
        2. Moderately trust.
        3. Do not trust very much.
        4. Do not trust at all.
    2. Scientists working to serve their employer or research funder.
        1. Strongly trust.
        2. Moderately trust.
        3. Do not trust very much.
        4. Do not trust at all.
6. For example, do you trust scientists who work at companies engaged in medical chemical manufacturing and agricultural supply and perform the tasks described below strongly, to some extent, not much, or not at all? (Choose one)
    1. Scientists working to serve the common good.

    1. Strongly trust.
    2. Moderately trust.
    3. Do not trust very much.
    4. Do not trust at all.
  2. Scientists working to serve their employer or research funder.
    1. Strongly trust.
    2. Moderately trust.
    3. Do not trust very much.
    4. Do not trust at all.

7. In general terms, regarding findings of accurate knowledge about the world, do you trust scientists strongly, to some extent, not much, or not at all? (Choose one) [Asked only in the second wave in March 2022]

   1. Strongly trust.
   2. Moderately trust.
   3. Do not trust much.
   4. Do no trust at all.

8. Have you become aware that you contracted the novel coronavirus disease (COVID-19) (if you do not want to answer, please do not answer and skip to the next question)? (Choose one)

   1. Yes.
   2. No.

9. Have you ever taken a vaccine other than those for the novel coronavirus disease? (Choose one)

   1. Yes.
   2. No.
   3. Do not want to answer.

10. In general, do you consider vaccination safe? (Choose one)

    1. Yes, I do.
    2. No, I do not.

11. In general, do you consider vaccination important? (Choose one)

    1. Yes, I do.

   2. No, I do not.

12. Do you trust vaccine licensing by the Ministry of Health, Labour and Welfare? (Choose one)

    1. Yes, I do.
    2. No, I do not.

13. Have you ever refused vaccination because of the danger of vaccination or suspicion about the efficacy of vaccination? (Choose one)

    1. Yes.
    2. No.

14. Have you postponed vaccination recommended by a doctor? (Choose one)

    1. Yes.
    2. No.

15. Whose opinions regarding vaccination do you trust or from whom did you seek such opinions? (Choose one)

    1. Family or close friends.
    2. Doctors.
    3. The internet.
    4. Other.
    5. Do not want to answer.

16. Do you believe that vaccination against the novel coronavirus disease should be mandated for Japanese nationals? (Choose one)

    1. Yes, it should.
    2. No, it should not.

17. Do you believe that the cost of vaccination against the novel coronavirus disease should be entirely covered by the government? (Choose one)

    1. Yes, it should.
    2. No, it should not.

18. Currently, do you have preexisting diseases under treatment (chronic cardiac diseases, kidney diseases, liver ailment, diabetes, etc.)? (If you do not want to answer, please skip to the next question without answering). (Choose one) [Asked only in the second wave in March 2022]

1. Yes.
2. No.

19. Have you taken the first dose of a vaccine against the novel coronavirus disease? (Choose one) [Asked only in the second wave in March 2022. If the answer was "No," respondents were directed to Question No. 27.]

    1. Yes, I have.
    2. No, I have not.

20. Have you taken the second dose of a vaccine against the novel coronavirus disease? (Choose one) [Asked only in the second wave in March 2022]

    1. Yes, I have.
    2. No, I have not.

21. Have you taken the third dose of a vaccine against the novel coronavirus disease? (Choose one) [Asked only in the second wave in March 2022]

    1. Yes, I have.
    2. No, I have not.

22. This is a question for respondents who have not taken the third dose of a vaccine against the novel coronavirus disease. Do you want to take the third dose of a vaccine against the novel coronavirus disease? If you have taken the third dose, please skip to the next question. (Choose one) [Asked only in the second wave in March 2022]

    1. Yes, I do.
    2. No, I do not.
    3. I do not know.

23. This is a question for respondents who have taken the first dose of a vaccine against the novel coronavirus disease (if you have not taken one, please skip to the next question without answering). Please tell us the manufacturer of the vaccine for your first dose. (Choose one) [Asked only in the second wave in March 2022]

    1. Pfizer.
    2. Moderna.
    3. AstraZeneca
    4. I do not remember it.

24. This is a question for respondents who have taken the second dose of a vaccine against the novel coronavirus disease (if you have not taken a second dose, please skip to the next question without answering). Please tell us the manufacturer of the vaccine for your second dose. (Choose one) [Asked only in the second wave in March 2022]

    1. Pfizer.
    2. Moderna.
    3. AstraZeneca
    4. I do not remember it.

25. This is a question for respondents who have taken a third dose of a vaccine against the novel coronavirus disease (if you have not taken a third dose, please skip to the next question without answering). Please tell us the manufacturer of the vaccine for your third dose. (Choose one) [Asked only in the second wave in March 2022]

    1. Pfizer.
    2. Moderna.
    3. AstraZeneca
    4. I do not remember it.

26. This is a question about the side effects of vaccination against the novel coronavirus disease. Have you experienced the side effect of the swelling of skin where the vaccine was injected ("COVID arm") after vaccination? [Asked only in the second wave in March 2022]

    1. Experienced after the first dose of vaccination.
    2. Experienced after the second dose of vaccination.
    3. Experienced after the third dose of vaccination.
    4. Have not experienced this symptom after the first, second, or third dose.

27. What is your marital status? (Choose one)

    1. Unmarried.
    2. Married.
    3. Divorced/Widowed.

28. How many children do you have? (Choose one)

    1. None.
    2. One.

3. Two.
4. Three.
5. Four.
6. Five or more.

29. Do you have siblings?

    1. Elder brother:
    2. Elder sister:
    3. Younger sister:
    4. Younger brother:

30. Are you employed? (Choose one)

    1. Yes, I am.
    2. No, I am not.

31. What is your employment status? (Choose one)

    1. Full-time regular worker.
    2. Part-time worker.
    3. Part-time casual worker.
    4. Dispatched employee/contracted employee.
    5. Board member of a company or other organization.
    6. Self-employed.
    7. Support for self-employed family member/Working on the side.
    8. Other.

32. Are you in a management position? (Choose one)

    1. No.
    2. Foreperson/Section leader/Principal.
    3. Assistant manager or equivalent.
    4. Department chief or equivalent.
    5. Division chief or equivalent.
    6. Other.
    7. I do not know.

33. How many employees does your employer have? (Choose one)

    1. 1–4.
    2. 5–29.
    3. 100–499.
    4. 500 or more.
    5. Government.

34. How much is your annual income? Please choose a bracket from those described below. (Choose one)

    1. 0.50 million Japanese yen or less.
    2. 0.50–0.99 million Japanese yen.
    3. 1.00–1.49 million Japanese yen.
    4. 1.50–1.99 million Japanese yen.
    5. 2.00–2.49 million Japanese yen.
    6. 2.50–2.99 million Japanese yen.
    7. 3.00–3.99 million Japanese yen.
    8. 4.00–4.99 million Japanese yen.
    9. 5.00 million Japanese yen or more.

35. How much is your household income? Please choose a bracket from those described below.

    1. 0.50 million Japanese yen or less.
    2. 0.50–0.99 million Japanese yen.
    3. 1.00–1.49 million Japanese yen.
    4. 1.50–1.99 million Japanese yen.
    5. 2.00–2.49 million Japanese yen.
    6. 2.50–2.99 million Japanese yen.
    7. 3.00–3.99 million Japanese yen.
    8. 4.00–4.99 million Japanese yen.
    9. 5.00–5.99 million Japanese yen.
    10. 6.00–6.99 million Japanese yen.
    11. 7.00–7.99 million Japanese yen.
    12. 8.00–8.99 million Japanese yen.

13. 9.00–9.99 million Japanese yen.
14. 10.00 million Japanese yen or more.

36. Please choose your highest degree (or education in which you are currently enrolled). (Choose one)

    1. Junior high school.
    2. High school.
    3. Vocational college.
    4. Two-year college.
    5. Technical college.
    6. Four-year college.
    7. Graduate school.

37. We would like to ask about your political attitudes. Which party do you usually support? (Choose one)

    1. Liberal Democratic Party.
    2. Constitutional Democratic Party.
    3. National Democratic Party.
    4. Clean Government Party.
    5. Japan Innovation Party.
    6. Japanese Communist Party.
    7. Social Democratic Party.
    8. Reiwa Shinsengumi (Reiwa Newly Selected Unit).
    9. Party to Protect Nationals who Do Not Pay NHK License Fee.
    10. Other.
    11. Do not support any party.

38. How satisfied are you with the current politics? (Choose one)

    1. Substantially satisfied.
    2. Modestly satisfied.
    3. Neither satisfied nor dissatisfied.
    4. Modestly dissatisfied.
    5. Substantially dissatisfied.

39. When individuals' interests conflict with those of the entire nation, which do you think should take precedence?

    (A) The entire nation's interests should be prioritized over individuals' interests.

    (B) Individuals' interests should be prioritized over the entire nation's interests.

    (Choose one)

    1. Close to A.
    2. Sort of close to A.
    3. Sort of close to B.
    4. Close to B.
    5. Do not agree with either.

40. Which of the following do you think about the role of government?

    (A) Even if governmental services such as social welfare deteriorate, a less costly small government is preferred.

    (B) Even if it is costly, a large government whose governmental services such as social welfare are excellent is preferred.

    (Choose one)

    1. Close to A.
    2. Sort of close to A.
    3. Sort of close to B.
    4. Close to B.
    5. Do not agree with either.

41. In both Japan and elsewhere, political positions are described in terms of "Left"–"Right". If your position is represented by this measure, where do you consider it to fall? From 0 (=the most right) to 10 (the most left), please pick one number that fits your position. (Choose one.)

42. If society as a whole is classified by social status, from highest (1) to lowest (10), which layer do you think you belong to? (Choose one)
